# *Amburana cearensis*: Pharmacological and Neuroprotective Effects of Its Compounds

**DOI:** 10.3390/molecules25153394

**Published:** 2020-07-27

**Authors:** Juliana Helena Castro e Silva, Rafael Short Ferreira, Erica Patricia Pereira, Suzana Braga-de-Souza, Monique Marylin Alves de Almeida, Cleonice Creusa dos Santos, Arthur Morgan Butt, Elisabetta Caiazzo, Raffaele Capasso, Victor Diogenes Amaral da Silva, Silvia Lima Costa

**Affiliations:** 1 Laboratory of Neurochemistry and Cell Biology, Department of Biochemistry and Biophysics, Institute of Health Sciences, Federal University of Bahia, Av. Reitor Miguel Calmon s/n Vale do Canela, 40100-902 Salvador, Bahia, Brazil; juhhmc@hotmail.com (J.H.C.eS.); rafael_short@hotmail.com (R.S.F.); epereira229@gmail.com (E.P.P.); suzanabrasilia@uol.com.br (S.B.-d.-S.); moniquemarylin27@gmail.com (M.M.A.d.A.); cleonicemev@gmail.com (C.C.d.S.); 2 School of Pharmacy and Biomedical Sciences, University of Portsmouth, St Michael’s Building, White Swan Road, Portsmouth, Portsmouth PO1 2DT, UK; Arthur.butt@port.ac.uk; 3 Department of Pharmacy, University of Naples Federico II, Via D. Montesano 49, 80131 Naples, Italy; elisabetta.caiazzo@unina.it; 4 Department of Agricultural Sciences, University of Naples Federico II, Via Università 100, 80055 Portici, Italy

**Keywords:** *Amburana cearensis*, pharmacological activities, central nervous system

## Abstract

*Amburana cearensis* A.C. Smith is an endemic tree from Northeastern Brazil used in folk medicine as teas, decocts and syrups for the treatment of various respiratory and inflammatory diseases, since therapeutic properties have been attributed to compounds from its stem bark and seeds. Numerous pharmacological properties of semi-purified extracts and isolated compounds from *A. cearensis* have been described in several biological systems, ranging from antimicrobial to anti-inflammatory effects. Some of these activities are attributed to coumarins and phenolic compounds, the major compounds present in *A. cearensis* seed extracts. Multiple lines of research demonstrate these compounds reduce oxidative stress, inflammation and neuronal death induced by glutamate excitotoxicity, events central to most neuropathologies, including Alzheimer’s disease (AD) and Parkinson’s Disease (PD). This review focuses on the botanical aspects, folk medicine use, biological effects and pharmacological activities of *A. cearensis* compounds and their potential as novel non-toxic drugs for the treatment of neurodegenerative diseases.

## 1. Introduction

Native plants of the legume family (Fabaceae) from the Brazilian Caatinga, a semi-arid biome, represent a large number of plants with ethnopharmacological properties for the treatment of various diseases [1]. Among them, *Amburana cearensis* A.C. Smith is a naturally occurring tree that is widely distributed in Northeastern Brazil and widely exploited in folk medicine [2]. The stem bark and seeds of *A. cearensis* have well-known therapeutic properties in the form of teas, decocts and syrups in the treatments of various respiratory conditions and toothaches, in addition to the use of the stem bark in a bath to relieve rheumatic and spasmodic pain [3,4,5], whilst in Bolivia, the stem bark is crushed and applied directly on the head for headache [6].

A range of pharmacological properties of extracts and isolated compounds from the different parts of *A. cearensis* have been reported in the literature. These properties include the antibacterial effect of a stem bark chloroform extract [7], the in vitro anti-malarial effect of a stem bark dichloromethane extract and a variety of isolated compounds with anti-falciparum, leishmanicidal and bactericidal activities [2]. Many studies attribute the action of these compounds present mostly in the stem bark and seed extracts to their anti-inflammatory potential [8,9], which stimulated research into its effects in experimental models of central nervous system (CNS) diseases associated with neuroinflammation. Notably, in vitro studies have reported neuroprotective effects of coumarin-rich extracts of *A. cearensis* seeds, especially a dichloromethane extract, which has been shown to be cytoprotective in brain cell lines and primary cell cultures [10,11] as well as a phenol glucoside from *A. cearensis* bark that protects against 6-hydroxydopamine toxicity [12]. These classes of compounds are known for their well characterized antioxidant actions and represent potential alternative therapies for neurodegenerative diseases. In this article, we present a review of the general aspects of *A. cearensis* botany and phytochemistry and the state of the art findings concerning *A. cearensis* pharmacological effects, including an overview of the latest findings on activities of *A. cearensis* products in CNS in vitro study models.

## 2. Botany

*A. cearensis*, popularly known in Brazil as “cumaru”, “cerejeira” or “imburana/umburana de cheiro”, is a tree that can grow to a height of up to 20 m. It has a natural distribution in the Brazilian “Caatinga” desert biome and also in the pluvial forest [2]. Although it is considered native to Brazil, it is also frequently found in other South American countries, such as Peru and Argentina [13].

*A. cearensis* was established by A.C. Smith (1940) as a botanical synonymous for *Torresea cearensis*, named by Freire Allemão in 1864. This species is one out of two species from the small *Amburana* genus of the Fabaceae family. *A. cearensis* stalk is a trunk and its inflorescences are made of both yellow and white flowers. Its leaves usually measure 10–15 cm containing leaflets that are usually 2 cm large. Its pods are dark and contain one or two black elliptical flying seeds that contribute to its pollination. A main characteristic of *A. cearensis* is its brown-reddish stem bark that is easily peeled off [5,14]. The plant bark has a high economic value due to its use in woodwork, which contributed to *A. cearensis* being classified as an endangered species in the past. Nowadays, it is considered almost endangered by the Brazilian National Center of Flora Conservation (Centro Nacional de Conservação de Flora) [2,15]. One study suggests that it should be used in the early stages of a program to reforest degraded seasonal forests and in plantation trials [16].

*A. cearensis* stem bark is used in folk medicine due to its curative properties, together with the use of its seeds in teas, decocts and syrups for diseases such as asthma, bronchitis, cough and toothache, whilst a bath of stem bark is used for rheumatic and spasmodic pain relief [5,13,17], particularly by indigenous natives [18].

## 3. Toxicology

Studies indicate that *A. cearensis* products are safe for consumption by both humans and other mammals. As described by Soares et al., 2007 [19], the chronic administration of an *A. cearensis* stem bark syrup (50 mg/mL) in twenty-four healthy male volunteers for four weeks demonstrated that this product is well tolerated according to biochemical, hematological, serological and clinical parameters (including electrocardiographic), and only two side effects were reported: dizziness (two volunteers) and nausea (one volunteer). The authors mentioned that many compounds were already isolated from *A. cearensis* trunk bark, including a coumarin that can alter prothrombin time and influence blood clot formation, and although it may induce acute side effects, such as hepatic necrosis, it has been shown to be safe overall, presenting only small fluctuations in renal and hepatic parameters. In another study by Leal et al., 2003 [20], the hydroalcoholic extract of *A. cearensis* stem bark at a dose of 500 mg/kg for up to 50 days did not show any teratogenicity and only altered alanine aminotransferase liver enzyme levels among all biochemical parameters measured; it also reduced the neutrophil counts in male rats and reduced creatinine concentration in female animals. A study by Costa-Lotufo et al., 2003 [21] showed that one hydroalcoholic extract of *A. cearensis* trunk bark, protocatechuic acid, can induce hemolysis in mouse erythrocytes, but the other extracts identified in the study did not (kaempferol, isokaempferide, amburoside A). Overall, these studies indicate that extracts of *A. cearensis* are non-toxic and well tolerated, presenting few side effects.

## 4. Chemical Constituents

Diverse pharmacological activities can be attributed to many secondary metabolites already characterized from several parts of the *A. cearensis* plant. From the trunk bark ethyl acetate extracts, Bravo B. et al., 1999 [22] identified two phenol glucosides in ethyl acetate extracts (amburoside A and amburoside B), in addition to coumarin (1-benzopyran-2-none) in the methylene chloride extract. Negri et al., 2004 [23] described predominantly phenolic compounds in the extracts from the stem bark, in particular coumarins (coumarin, dihydrocoumarin and scopoletin), phenylpropanoids (e.g., *trans*-methyl-3,4-dimethoxycinnamic acid), benzoic acids (e.g., methyl-3-methoxy-4-hydroxybenzoic acid), simple phenols (e.g., catechol and guaiacol) and the anthraquinone chrysophanol. Triterpenoids (e.g., lupeol and amyrins), steroids (e.g., γ-sitosterol), aliphatic esters (e.g., methyl palmitate) and other minor compounds were also detected in this study. Using the dry stem bark chloroform extract, Sá et al., 2014 [7] identified other different components: a benzenoid, 4-methoxy-3-methylphenol, together with bicyclic monoterpenes, such as α-pinene and β-pinene and 4-hydroxy-3-methoxybenzoic acid. The ethanolic extract of stem bark obtained by Canuto and Silveira, 2010 [3] provided coumarin as the major compound, as well as two phenolic acids (vanillic acid and protocatechuic acid), amburoside A (a phenol glucoside), a mixture of glycolyzed phytosterols (β-sitosterol and stigmasterol) and five flavonoids (afrormosin, isokaempferide, kaempferol, quercetin and 4′-methoxy-fisetin).

Proteins that represent protection mechanisms for the plants, especially the seeds, can be a source of molecules as tools for studying proteinase functions. Using *A. cearensis* seeds, authors have isolated TaTI (Torresea acreana trypsin inhibitor), a new member of the Bowman-Birk trypsin inhibitor family [24,25]. Furthermore, from *A. cearensis* seed hexane suspension, Rodrigues et al., 2008 [26] purified coumarin, using supercritical carbon dioxide and a semi-continuous flow apparatus. Using the same part of the plant, Pereira et al., 2017a [10] obtained an ethanolic extract (ETAC) and performed different partitions with hexane (EHAC), dichloromethane (EDAC) and ethyl acetate (EAAC); coumarin and its 3-methyl derivative, fatty acid esters (methyl octanoate, methyl hexadecanoate, methyl 13-trans-octadecanoate) and phytosterols like γ-sitosterol were identified in all partitions except in EAAC. Some of these metabolites are also present after germination, as described by Canuto and Silveira, 2010 [3]; using an ethanolic extract of both the ground parts and xylopodiums of young specimens, the authors purified nine compounds after dichloromethane partition, silica gel chromatography and other treatments, namely coumarin, isokaempferol, amburoside A, amburisode B, vanillic acid, p-hydroxybenzoic acid, aiapin, protocatechuic acid and *E*-(*o*)-glycosylated coumaric acid.

Coumarins appear to be the main secondary metabolites identified in *A. cearensis*. These compounds are natural benzopyrene derivatives that display important biological roles in vegetables and microorganisms. Coumarins are pharmacologically known as antioxidants and anti-inflammatory drugs [27,28,29] and are naturally present in various parts of the plant, controlling cell growth and energetic functions. Natural coumarins are synthesized through the shikimic acid pathway, and its first isolation dates to 1820 by Vogel. Ever since, a wide range of biological effects have been observed also from its synthetic derivatives, probably due to the simple structure of coumarins [30]. Antimicrobial, anti-inflammatory and antitumoral activities have been described for coumarins [21,22,23], but their more recognized biological activity is as an anticoagulant [24].

## 5. Biological Activities

Many biological effects of *A. cearensis* extracts and isolated compounds have been described (Table 1). Sá et al., 2014 [7] demonstrated the bacteriostatic activity of the stem bark chloroform extract on *Pseudomonas aeruginosa* and *Bacillus cereus* strains, with a minimum inhibitory concentration (MIC) of 6900 µg/mL. In addition, this work describes the broad-spectrum bactericidal effect of the synthetic 2-methoxy-4-methylphenol (MIC 215 to 431 µg/mL), a purchased compound analogue to 4-methoxy-3-methylpoliphenol, contained in the extract.

Recently, Oliveira et al., 2020 [31] have shown a potential synergistic antibacterial effect of total protein extract of *A. cearensis* seeds and three fractions obtained from precipitation with different ammonium sulfate concentrations against *Staphylococcus aureus* and *Escherichia coli* strains, together with known antibacterial drugs (norfloxacin, penicillin and gentamicin). The crude extract, together with the three antibiotics, inhibited *S. aureus* and *E. coli* growth. In comparison, the three fractions presented non-homogeneous results. Additionally, the three ammonium sulfate fractions were able to inhibit the activity of the serine protease trypsin, which, in plants, has a key role in regulating proteolytic effects and modulating apoptosis, and is a representative of the plant’s defense system.

**Figure 1:**
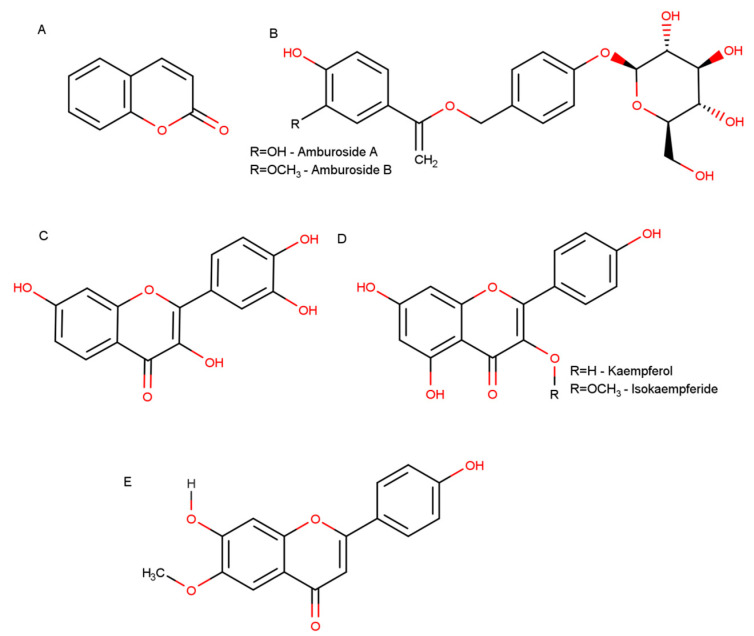
Representative chemical structures of *A. cearensis* bioactive compounds. (**A**) Coumarin (1,2-benzopyrone); (**B**) phenol glucosides amburoside A and amburoside B; (**C**) fisetin; (**D**) flavonoids kaempferol and isokaempferide; (**E**) flavonoid afrormosin.

In another study, Bravo B. et al., 1999 [22] reported the in vivo anti-malarial effect of dichloromethane stem bark extract against a *Plasmodium falciparum* strain sensitive to chloroquine in mice. The extract inhibited 50% of the parasite activity at a concentration (IC50) of 9 µg/mL. Isolated compounds from the extract, coumarin (1-benzopyran-2-ona, Figure 1A) and amburoside A (Figure 1B), at a dose of 50 mg/kg/day, administered for 4 days, also reduced the parasitemia by 25 and 24% in mice, respectively. Moreover, in vitro, coumarin itself, at a concentration of 50 μg/mL, could reduce the viability of *Leishmania amazonensis*, *L. braziliensis* and *L. donovani* at a concentration of 50 μg/mL.

Farias et al., 2010 [32] described *A. cearensis* aqueous extract as being 100% toxic for *Aedes aegypt* mosquito larvae, a species that is a vector for a variety of viruses with significant epidemiology in Brazil. Toxicity was observed within 3 h from a concentration of 24.75 ± 1.31 mg/mL of soluble solids, obtaining a LC50 = 8.10 ± 0.27 mg/mL. Using phytochemical rapid analysis of the extracts, the authors detected the presence of tannins, phenols and flavones. This study also mentions the extracts have trypsin inhibition activity, with 12.23 ± 0.29 g of trypsin being inhibited per kg of extract. Together, these findings suggest that *A. cearensis* extract contains molecules that are involved in plant defense and can act as larvicidal via inhibition of proteases. Interestingly, the extract did not present acute toxicity for mice (30 mL/Kg).

Two studies mention the potential of *A. cearensis* leaf ethanolic extract in modulating the ovarian follicle cycle. Barberino et al. (2015) [33] tested the extract (0.1–0.4 mg/mL) in cultured ovine ovaries and showed that it maintains ovary viability in the absence of supplements for up to 18 days after culture (particularly 0.1 mg/mL). Gouveia et al., 2016 [34] tested the leaf extract in caprine ovaries and found the extract improves follicle growth and survival in supplemented media (particularly 0.2 mg/mL).

*A. cearensis* compounds have also been tested for anticancer activities. In a study by Costa-Lotufo, 2003 [21], kaempferol, isokaempferide, amburoside A and protocatechuic acid, isolated from *A. cearensis* trunk bark methanolic extract, have shown cytotoxic effects against cancer cell lines B-16 (murine skin cancer), HCT-8 (human colon cancer), MCF-7 (human breast cancer) and CEM and HL-60 (leukemia), using the MTT (3-(4,5-dimethyl-2-thiazolyl)-2,5-diphenyl-2H-tetrazolium bromide) viability assay. The authors have also tested the antiproliferative activity in a sea urchin model, and it was demonstrated that isokaempferide and kaempferol inhibited sea urchin egg development, as well as induced cytotoxicity in tumor cell lines, but in this assay isokaempferide was more potent than kaempferol.

The assortment of biological activities of *A. cearensis* may justify its popular ethnopharmacological use as an anti-inflammatory and analgesic agent. Leal et al., 2008 [35] have shown that both 25 and 50 mg/kg of amburoside A isolated from *A. cearensis* trunk bark reduced liver necrosis and the inflammatory infiltrate in the liver parenchyma in a carbon tetrachloride model of induced liver injury. Furthermore, this compound normalized catalase activity and decreased the induced lipid peroxidation.

Leal et al., 1997 [8] demonstrated an antiedematogenic effect of *A. cearensis* stem bark hydroalcoholic extract (HAE), its flavonoid fraction and coumarins, in a carrageenan and dextran-induced rad paw edema model; coumarins (10 and 20 mg/kg) and HAE (100 and 200 mg/kg) induced a dose-dependent reduction of paw edema and inflammatory exudates, whilst HAE also reduced the number of abdominal contractions induced by acetic acid by up to 49%. In the same study, HAE (100, 200 and 400 mg/kg) and coumarins (5 and 10 mg/kg) decreased nociception in male Swiss mice by increasing the latency time in a hot-plate test in a dose-dependent manner. In another study by Leal et al. (2000) [36], with a view to investigating the pharmacological use for respiratory tract diseases, the antinociceptive and anti-inflammatory activities of hydroalcoholic extracts from *A. cearensis* stem bark extract were reproduced, and the extract also showed bronchodilator activity in an acetic acid-induced writhing model in mice. A phytochemical study showed that the extract tested positive for the presence of coumarin, flavonoids, saponins and tannins.

Two other works corroborate these effects of *A. cearensis* compounds. Lima et al., 2013 [14] have shown an antiedematogenic effect of the stem bark aqueous extract (10% and 20%) in a carrageenan-induced paw edema model. In the same study, this extract presented mutagenic effects, which might amplify the necessity of toxicological assays on the products of this species to make its use safe. Moreover, Leal et al., 2003 [37] isolated coumarin (5 mg/kg) and a flavonoid fraction (FF) (20 mg/kg) from HAE (150 mg/kg), and oral administration of both compounds in Wistar rats reduced cutaneous vascular permeability induced by serotonin; the HAE also reduced vascular permeability induced by histamine. The authors also demonstrated that HAE (200 and 400 mg/kg), FF (10, 20 and 40 mg/kg) and coumarin (20 and 40 mg/kg) inhibited neutrophil and leukocyte migration into the peritoneal cavity induced by carrageenan and *N*-formylmethionine-leucyl-phenylalanine. The HAE and FF compounds were also able to induce trachea muscle relaxation in Guinea pig, which might suggest both anti-inflammatory and bronchodilator effects.

In this light, afrormosin, a flavonoid isolated from *A. cearensis* trunk bark, reduced the myeloperoxidase release by human neutrophils and other neutrophil responses after the cells were stimulated by phorbol 12-myristate-13-acetate (PMA). In this model, analysis of the cell suspension demonstrated that afrormosin reduced neutrophil degranulation (IC50 = 0.37 µM) and TNF-α levels up to 44% and showed an antioxidant effect from at least 83.8 µM concentration [38].

Another study by Marinho et al., 2004 [9] showed paw edema induced by ovalbumin sensibilization was reduced using trunk bark HAE administrated orally (400 mg/kg) or intraperitoneally (100 mg/kg), as well as extracted coumarin (10 and 20 mg/kg) administrated intraperitoneally. In this work, both HAE and coumarin specifically reduced anti-ovalbumin immunoglobulin levels. Moreover, HAE (100 and 200 mg/kg intraperitoneal) also inhibited acetic acid-induced vascular permeability.

## 6. Neuroprotective Activities of *A. cearensis* Compounds

Neurodegenerative diseases (ND) result in chronic and progressive degeneration of neurons, which leads to cognitive, neuropsychiatric, behavioral and/or motor symptoms. These diseases are often related to ageing and the inability of both neurons and glial cells to proliferate and compensate for insults and cell death. Currently, ND represent a major public health priority around the globe as life expectancy increases, mainly in European and North American countries. Alzheimer’s disease (AD) is the most common ND, with the World Health Organization (WHO) estimating in 2012 that 35.6 million people worldwide were living with dementia (with AD accounting for 60–70%) and that this number would double by 2030 and triple in 2050, bringing more social and economic impacts than cancer [39,40]. The second most common ND is Parkinson’s disease (PD), with 6.1 million people worldwide having this disease in 2016 [41,42]. In addition to ND, stroke is another important CNS pathology that stands out for alarming statistical data. Data reported in 2011 show that approximately 800,000 primary (first-time) or secondary (recurrent) strokes occur each year in the US; of these strokes, approximately 87% are ischemic infarctions, 10% are primary hemorrhages and 3% are subarachnoid hemorrhages [43].

Despite extensive research, particularly over the last 30 years, the specific causes of neuron loss in ND remain unclear and are likely to be multifactorial. There are multiple hypotheses for the causes of neuron loss in different neuropathologies, many of which are complementary. For example, it is well known that misfolded proteins and peptide aggregates can lead to neural toxicity, such as α-synuclein in PD and β-amyloid in AD [44]. In PD, it is common knowledge that dopaminergic neurons degenerate, especially in the substantia nigra of the striatum. These cells contain neuromelanin, a natural endogenous product formed by the oxidation of dopamine to aminochrome, a more stable molecule [45,46,47]. Studies have demonstrated that aminochrome induces the formation of neurotoxic oligomers that can stabilize toxic alpha-synuclein protofibrils [48,49]. Neurotoxicity is also related to the presence of α-synuclein aggregates in Lewy bodies in the neuron cytoplasm, which contributes to mitochondrial damage and oxidative stress [50,51,52]. In AD, the accumulation of β-amyloid peptides in the extracellular space and neurofibrillary tangles also leads to toxicity to neurons, mainly cholinergic neurons, of the frontal cortex, parietal lobe, frontal lobe, limbic structures and the cortex-hippocampal connections. In addition, there may be an accumulation of intracellular misfolded tau proteins [53,54,55].

Oxidative stress is another common factor in ND, for example accelerating dopamine degradation and α-synuclein toxicity in PD [48,56], and might be considered as the main hypothesis for neuronal death, because it impairs the cellular antioxidant defense ability [57,58]. Reactive oxygen species (ROS) followed by a decrease in the potential for cellular antioxidant defense is one of the main hypotheses of mechanisms for triggering apoptosis of nigrostriatal neurons in PD and patients with PD having higher blood concentrations of oxidative markers [59,60]. Oxidative stress is also considered the earliest event in the appearance of AD [52,58,61]. In addition, oxidative stress can impair the reduced and oxidized glutathione antioxidant system (GSH/GSSG) [62], enhance basal lipid peroxidation and increase the activity of mitochondrial superoxide dismutase [63]. Mitochondrial complex IV is also affected in AD and, together with astrocyte and microglia activation and neuronal death, can lead to the production of pro-inflammatory factors that can start or persist degeneration [64,65].

Glutamate-mediated excitotoxicity is a further factor in the etiology of neuronal death in ND and stroke [66,67,68]. Glutamate is the most abundant neurotransmitter in the CNS and under pathological conditions can accumulate extracellularly, which leads to the process known as “glutamatergic excitotoxicity”. Besides being involved in acute brain ischemia, this process leads to metabolic changes that are known as a final and convergent pathway in neuronal death among many ND [69,70]. Excessive levels of glutamate in the CNS, resulting from hypoxia, physical trauma and/or a failure in the reuptake of this neurotransmitter by astrocytes, can trigger signaling cascades that culminate in excessive Ca^2+^ entry into cells and the activation of cell death cascades, deregulation of mitochondrial membrane potentials and oxidative stress [71,72,73]. These events are related to neuronal death in both experimental AD and PD [53,74].

Neuroinflammation also plays a critical role in neurodegeneration. Inflammation is a physiologically important mechanism for containing insults. However, when the inflammatory response is exacerbated in the CNS, it can be irreversible for neural cells. Neurodegenerative and neurological diseases can often present reactive gliosis as a pathological finding, where glial cells, mostly astrocytes and microglia, respond to insults by secreting pro-inflammatory factors and changing their phenotype in a way that restricts a lesion [75,76,77,78]. The secretion of molecules, such as interleukins, chemokines and reactive species of oxygen and nitrogen by microglia and astrocytes, can lead to their chronic activation and prompt them towards a toxic phenotype, which in turn promotes more neuronal death. Microglia, astrocytes and neurons are able to release these molecules, which might be involved in the initiation and progression of neurodegeneration [79,80]. In post mortem tissue, activated microglia were found in the substantia nigra in PD, and surrounding senile plaques and tau protein tangles in AD [81]. Microglia are the intrinsic immune cells of the CNS and exhibit multiple states of activation in response to pathology. Although microglial activation states display a high degree of heterogeneity, two distinct polarized microglial phenotypes have been described in the literature, based on expression of specific proteins and cytokines/chemokines, termed pro-inflammatory M1 microglia and regulatory M2 microglia. The more pro-inflammatory microglial phenotype is characterized by production of cytokines such as IL-1β and TNF, which is essential for controlling tissue damage and cleaning cell debris. The switch to a more regulatory M2 phenotype can be characterized by the secretion of factors such as IL-4 and IL-10 that promote brain repair [82,83]. For demyelinating diseases, such as multiple sclerosis (MS), there is an infiltration of Th1 lymphocytes from the immune system into the nervous system that recognize myelin and start a cytokine-mediated reaction that stimulates microglia and infiltrated macrophages to produce IL-12 and ROS, culminating in axonal and, consequently, neuronal death [80,84,85]. There is now an abundance of evidence from microglial transcriptomic and proteomic profiles that characterizing microglia as being exclusively in an M1 or M2 state is over simplistic and this classification should not be used going forward. However, the M1/M2 terminology remains in use as an indicator of microglial function, and it is evident that modulating the inflammatory functions of microglia is an important therapeutic strategy in ND.

Frequently, anti-inflammatory drugs are described to have neuroprotective effects [86,87,88,89], as demonstrated in models of ND. Hence, antioxidant substances, such as coumarins, are of interest in the research on therapeutic and preventive treatments for ND. In addition, other phenolic and benzopyran derivative compounds, such as flavonoids, are well known as anti-inflammatory drugs that can modulate the expression of cytokines and inflammatory transcription factors, such as NF-κB, nitric oxide synthase (iNOS) and, consequently, nitric oxide expression [27,30,90]. Flavonoids are also neuroprotective both in vitro and in vivo and are capable of controlling cytokine, chemokine and ROS expression [10,11,91,92,93].

There is positive evidence for the pharmacological application of *A. cearensis* compounds in ND models. The *A. cearensis* stem bark extract at a concentration of 2.3 mg/mL has been shown to effectively inhibit acetylcholinesterase activity in microplate and thin-layer chromatography assays [94]. Acetylcholinesterase inhibitors are the most commonly used drugs for treating AD and other types of dementia [95]. Although they cannot stop the progression of the disease, acetylcholinesterase inhibitors act by increasing brain levels of acetylcholine, which are decreased in AD due to degeneration of cholinergic neurons, especially in the forebrain [96].

Leal et al., 2005 [12] examined the effects of amburoside A from *A. cearensis* trunk bark in an in vitro model of PD, using 6-hydroxydopamine (6-OHDA) in mesencephalic cells from Wistar rat embryos. 6-OHDA is used as an in vitro and in vivo model for PD because it is an endogenous compound formed by dopamine oxidation that induces dopaminergic neuronal degradation and is involved in the pathogenesis of PD. 6-OHD has been shown to induce many of the molecular findings in PD in vitro and in vivo, such as oxidative stress, mitochondrial damage, neuroinflammation and cell death via apoptosis [97,98,99]. Using the MTT toxicity assay, Leal et al., 2005 [12] showed that amburoside A was not toxic in any of the concentrations tested and that pre-treatment at concentrations starting from 0.5 µg/mL protected the cell culture from reduced viability induced by 6-OHDA toxicity. Amburoside A at concentrations of 0.1–10 µg/mL also decreased cell culture nitric oxide production when compared to 6-OHDA. Furthermore, the increase in the levels of thiobarbituric acid reactive substances (TBARS) induced by 6-OHDA, an indication of lipid peroxidation [100], was inhibited by pre-treatment with amburoside A. The findings together show that amburoside A is a neuroprotective agent for cell culture of mesencephalon/midbrain, an important region for PD [88,101], and, as for other phenolic compounds [92], can act as an anti-inflammatory and antioxidant compound capable of reducing reactive nitrogen species [102].

*A. cearensis* compounds have been shown to have neuroprotective effects in ND-related models of glutamatergic excitotoxicity. In a work by Pereira et al. (2017a) [10], extracts from *A. cearensis* seeds protected a neuronal PC-12 cell line from neurotoxicity when exposed to high concentrations of glutamate (1 mM); the authors tested four different partitions: an ethanolic extract (ETAC) and its partitions with hexane (EHAC), dichloromethane (EDAC) and ethyl acetate (EAAC). None of these extracts were neurotoxic at a range of concentrations tested (0.1 to 2000 µg/mL), except EHAC, which reduced cell viability at the highest concentration tested (1000 µg/mL) after 72 h of treatment. The EDAC was characterized by Pereira et al., 2017a [10] as having the richest extract in coumarins and fatty acids esters, such as methyl hexadecanoate, ethyl hexadecanoate and methyl 13-octadecanoate. In another study by Pereira et al., 2017b [11], the effects of EHAC, EDAC and EAAC were tested in a more complex in vitro model, in cerebellum primary cultures, which contain neurons, astrocytes, microglia and progenitor cells. *A. cerarensis* extracts showed no toxicity to cerebellar cells in concentrations ranging from 0.1 to 100 µg/mL for up to 72 h treatments and protected the cells from neuronal degeneration induced by high levels of glutamate. The EDAC fraction also prevented morphological changes induced by glutamate in neurons and astrocytes; the latter displayed typical astrogliosis induced by glutamate, with an increase of glial fibrillary astrocyte protein (GFAP) and glutamine synthetase (GS) expression, which suggested that *A. cearensis* seed extracts may act by controlling astrocytic metabolism and neuroprotective properties.

As described above, most of the molecules present in bioactive *A. cearensis* extracts and fractions are phenolic compounds, such as coumarin, phenol glucosides and flavonoids (Figure 1). These compounds are versatile molecules that have been tested in CNS models and are known for their neuroprotective effects [92]. In vitro and in vivo studies show that phenolic compounds, including fisetin and coumarins, present in *A. cearensis* can act as antioxidants and reduce oxidative stress and mitochondrial damage [103,104,105,106], act as an anti-inflammatory by reducing the levels of pro-inflammatory cytokines that can initiate and persist chronic inflammation and increasing the levels of regulatory cytokines [107,108,109], reduce misfolded protein cytotoxicity [110,111], act as neurogenic drugs [93,112] and modulate glutamate cytotoxicity [93,113,114]. To sum up, these compounds from *A. cearensis* can act in a multitargeted way to reduce neurodegeneration and improve neural cell function and viability.

## 7. Conclusions

*Amburana cearensis* A.C. Smith is an endemic tree from Northeastern Brazil that is used in folk medicine. The folk use of *Amburana cearensis* has been justified by scientific research describing the pharmacological properties of the compounds present in extracts from several parts of this plant. Importantly, there is growing evidence that compounds extracted from the seeds of *Amburana cearensis* are a source of novel neuroprotective compounds, including coumarin, phenol glucosides, such as amburoside A, and flavonoids, and can target multiple neurodegenerative diseases, particularly because of their ability to reduce oxidative stress, inflammation and neuronal death induced by glutamate excitotoxicity.

## Figures and Tables

**Table 1:** Isolated compounds from different extracts and parts of *A. cearensis* and related biological activities.

Author (Year)	Plant Part	Extract	Isolated Compounds	Activity
Leal et al., 1997	Bark	Hydroalcoholic extract	Coumarin; isokaempferol, flavonoids (fraction).	Hydroalcoholic extract and coumarin: anti-nociceptive; anti-inflammatory
Bravo B. et al., 1999	Bark	Dichloromethane extract	Coumarin, Amburoside A, Amburoside B.	Extract: anti-plasmodium Coumarin: leishmanicidal, bactericidal and antimalarial Amburoside A: antimalarial
Leal et al., 2000	Bark	Hydroalcoholic extract	-	Antinociceptive, anti-inflammatory and bronchodilator
Costa-Lotufo, 2003	Bark	Methanolic extract	kaempferol, isokaempferide, amburoside A and protocatechuic acid	Isolated compounds: antiproliferative and antioxidant
Leal et al., 2003a	Bark	Hydroalcoholic extract	Coumarin; 3,4,5-trihydroxi-benzoic acid; isokaempferol; fisetin and a biflavonoid	Extract, coumarin and flavonoid fraction: anti-inflammatory; (bronchodilator).
Marinho et al., 2004	Bark	Hydroalcoholic extract	Coumarin	Immunomodulation of specific antibodies; vascular permeability reduction.
Leal et al., 2005	Bark	Ethanolic extract	Amburoside A	Amburoside A: neuroprotective
Leal et al., 2008	Bark	Ethanolic extract	Amburoside A	Hepatoprotective and anti-inflammatory
Farias et al., 2010	Seeds	Aqueous extract	-	Toxicity against *Aedes aegypti* larvae
Lima et al., 2013	Seeds	Aqueous extract	-	Anti-edematous
Lopes et al., 2013	Bark	Ethanolic extract	Afrormosin	Afrormosin: inhibition of neutrophil responses
Sá et al., 2014	Bark	Chloroform extract	-	Extract: bacteriostatic
Barberino et al., 2015	Leaves	Ethanolic extract	-	Extract: improves in vitro development of ovine secondary follicles
Gouveia et al., 2016	Leaves	Ethanolic extract	-	Improves in vitro development of caprine preantral follicles
Pereira et al., 2017a	Seeds	Ethanolic extract and fractions	-	Ethanolic extracts and fractions: neuroprotective (glutamate-induced excitotoxicity) to cell lines
Pereira et al., 2017b	Seeds	Ethanolic extract and fractions	-	Ethanolic extracts and fractions: neuroprotective (glutamate-induced excitotoxicity) to primary culture
Oliveira et al., 2020	Seeds	Protein extract and fractions	-	Synergistic antibacterial effect against *S. aureus* and *E. coli*; trypsin activity inhibitor.
